# Establishment of one-step SYBR green-based real time-PCR assay for rapid detection and quantification of chikungunya virus infection

**DOI:** 10.1186/1743-422X-7-13

**Published:** 2010-01-21

**Authors:** Phui San Ho, Mary Mah Lee Ng, Justin Jang Hann Chu

**Affiliations:** 1 School of Applied Science, Republic Polytechnic, 9 Woodlands Avenue 9, 738964, Singapore; 2 Department of Microbiology, Yong Loo Lin School of Medicine, 5 Science Drive 2, National University Health System, National University of Singapore, 117597, Singapore

## Abstract

Chikungunya virus (CHIKV) is a mosquito-borne alphavirus and one of the prevalent re-emerging arbovirus in tropical and subtropical regions of Asia, Africa, and Central and South America. It produces a spectrum of illness ranging from inapparent infection to moderate febrile illness as well as severe arthralgia or arthritis affecting multiple joints. In this study, a quantitative, one-step real-time SYBR Green-based RT-PCR system for the non-structural protein 2 (nsP2) of CHIKV that can quantify a wide range of viral RNA concentrations was developed. Comparisons between the conventional semi-quantitative RT-PCR assay, immunofluorescence detection method and the one-step SYBR Green-based RT-PCR assay in the detection of CHIKV infection revealed much rapid and increase sensitivity of the latter method. Furthermore, this newly developed assay was validated by *in vitro *experiments in which ribavirin, a well-known RNA virus inhibitor, showed a dose-dependent inhibition of virus replication on cells that was assessed by viral infectivity and viral RNA production. Our results demonstrate the potential of this newly developed one-step SYBR Green I-based RT-PCR assay may be a useful tool in rapid detection of CHIKV and monitoring the extent of viral replication possibly in patients' samples.

## Findings

Chikungunya virus disease or chikungunya fever, is a viral disease transmitted to humans by the bite of infected *Aedes_aegypti *(yellow fever mosquito) *and Aedes albopictus *(tiger mosquito) [1]. Chikungunya virus (CHIKV) is a member of the genus alphavirus, in the family *Togaviridae *[2]. CHIKV contains a non-segmented monomeric, positive-sense RNA genome that is approximately 12 Kb. CHIKV genome is arranged in the order of 5' cap-nsP1-nsP2-nsP3-nsP4-(junction region)-C-E3-E2-6K-E1-poly(A)3' where nsP1 is RNA-capping enzyme, nsP2 contains the protease, triphosphatase, NTPase and helicase, nsP3's function is currently unknown and nsP4 contains RNA dependant RNA polymerase. The capsid (C) forms the nucleocapsid that enclosed the viral RNA and E1, E2 as well as E3 encodes for the envelope protein of the virus [2].

CHIKV was first isolated from the blood of a febrile patient in Tanzania in 1953 [3,4], and has since been identified repeatedly in west, central and southern Africa and many areas of Asia, and has been cited as the cause of numerous human epidemics in those areas since that time [5].

CHIKV produces an illness in humans that is often characterized by a sudden onset of fever, headache, fatigue, nausea, vomiting, rash, myalgia, and severe arthralgia (joints pain) [6]. Symptoms are generally self-limiting and last 1 to 10 days. However, arthralgia may persist for months or years [7]. These clinical symptoms mimic those of dengue fever and malaria, and therefore, many cases of Chikungunya fever are misdiagnosed as dengue virus infections [8]. At present, there is no vaccine or anti-viral therapy available against CHIKV infection, but analgesics and anti-inflammation medication are usually used to reduce the swelling and pain.

Routine assays for the detection and quantification of CHIKV are essential tools in research areas addressing the replication biology of CHIKV, clinical diagnostic applications and the development of effective anti-viral strategies. Virus isolation by cell culture and followed by quantification via viral plaque assay remains the "gold standard" although it has the disadvantage that longer than 7 days is usually required to complete the test. Other biochemical and immunoassays, such as ELISA-based techniques [9] for the quantification of CHIKV envelope proteins are often expensive and require extensive sample dilution, because they are linear only over a limited range of antigen concentration. In addition, reverse transcription-PCR strategies to detect and quantify CHIKV genomes rely on sequence-specific primer design and require RNA isolation procedures which make it expensive and labour intensive. Recently, several studies have illustrated the application of real-time (RT) PCR technology or RT-loop-mediated isothermal amplification assay in the detection of CHIKV infection [10,11]. The RT- PCR assay has many advantages over conventional reverse transcription-PCR methods, including rapidity, quantitative measurement, lower contamination rate, higher sensitivity, higher specificity, and easy standardization. Thus, quantitative RT-PCR assay might eventually replace virus isolation and conventional reverse transcription-PCR as the new gold standard for the rapid detection, quantification and diagnosis of CHIKV infection.

In this study, we attempt to develop a rapid and reliable one-step quantitative RT-PCR assay based on SYBR Green DNA dye-binding fluorophore that detect CHIKV infection. SYBR Green DNA-based RT-PCR systems are good alternative to fluorescent probe-based RT-PCR techniques and are based on its ability to produce a 100-fold increase of fluorescence when bound to double-stranded DNA. The binding of SYBR Green to nucleic acid is not sequence-specific and the fluorescent signal produced when in complex with DNA is directly proportional to the length and amount of DNA copies synthesized during the reaction hence making this technique very precise and sensitive. Given the popularity of the SYBR Green-based systems and their cost efficiency, the SYBR Green chemistry has been adapted in this study to develop an efficient one-step RT-PCR assay for CHIKV detection and quantification.

The additional advantage of utilizing SYBR Green-based real-time RT-PCR is that it is relatively easy to design and test primer pairs that are suitable for RT-PCR analysis. In this study, primers were selected based on highly conserved regions of CHIKV genome. After nucleotide sequence alignment using DNASTAR Lasergene Version 7.2 software (DNASTAR) of the CHIKV strains for which the entire genome sequences are available in GenBank (GenBank accession no: AF490259 of Ross reference strain, EU703762.1 of Malaysia strain, EF027140.1 of Indian strain, AM258995.1 of Reunion strain and EF452494.1 of USA strain), the potential target regions were identified in the regions of E1, nsP1, nsP2, and nsP4 genes. More than 20 sets of primer pairs were synthesized and screened for initial evaluation of their specificity and sensitivity (Data not shown). Among these primers tested, a set of specific primer pairs in the nsP2 gene region of CHIKV genome was found to be most sensitive. The primers were also designed to take into account of possible single nucleotide mismatches among strains and to avoid primer-dimer formations. The final sequences and the genomic location of the selected nsP2 primers are shown in Table S1, Additional file 1. These primers sequences showed 100% identity with the reference strain-ROSS, African strain S27 and the viral clusters from Malaysia. High identity (only single nucleotide mismatch) with the clusters from India, Mauritius and Reunion was noted and most likely capable to detect of these viruses (Table S1, Additional file 1.). However, the primers showed several mismatches with nsP2 sequence from West African, Senegal strain 37997 (GenBank accession no. AY726732.1), it is likely to expect that our current system would not recognize this strain, although it has not been tested.

CHIKV isolates from the Singapore local outbreaks were kindly provided by Dr Ooi Eng Eong (Duke-NUS Medical School, Singapore) and Dr Raymond Lin (National Public Health Laboratory, Ministry of Health, Singapore). CHIKV was propagated in Vero and C6/36 cells and infectious virus titers were determined by plaque forming assay using BHK cells in the Biosafety Level 2 facilities. Supernatants were obtained 3 days after virus inoculation and stored at -80°C. Viral RNA was extracted (Qiamp viral RNA kit; Qiagen, Germany) from the virus supernatant, titrated with plaque forming assays [12], and serial diluted accordingly to the plaque forming units (PFU) per ml. One-step SYBR green-based RT-PCR was optimized and carried out using the SYBR Green Quantitative RT-PCR Kit (SIGMA-ALDRICH^®^, QR0100) in the ABI PRISM 7000 RT-PCR system. CHIKV samples were assayed with a concentration (250 nM) of each nsP2 primers in a 1× final concentration of SYBR green Taq Ready Mix for Quantitative TR-PCR (1× *Taq *DNA polymerase, 10 mM Tris-HCl, 50 mM KCl, 3.0 mM MgCl2, 0.2 mM dNTP, stabilizers) and 1× reference Dye. The RT-PCR conditions for the one-step SYBR green I RT-PCR consist of a 20-min reverse transcription step at 44°C and then 2 min of Taq polymerase activation at 94°C, followed by 40 cycles of PCR at 94°C for15 seconds (denaturation), 60°C for 1 min (annealing and extension). Amplification graphs were checked for the Ct value of the PCR product. The Ct value represented the cycle by which the fluorescence of a sample increased to a level higher than the background fluorescence in the amplification cycle. An amplified product of 107-bp fragment was obtained from the nsP2 primer set.

**Figure 1:**
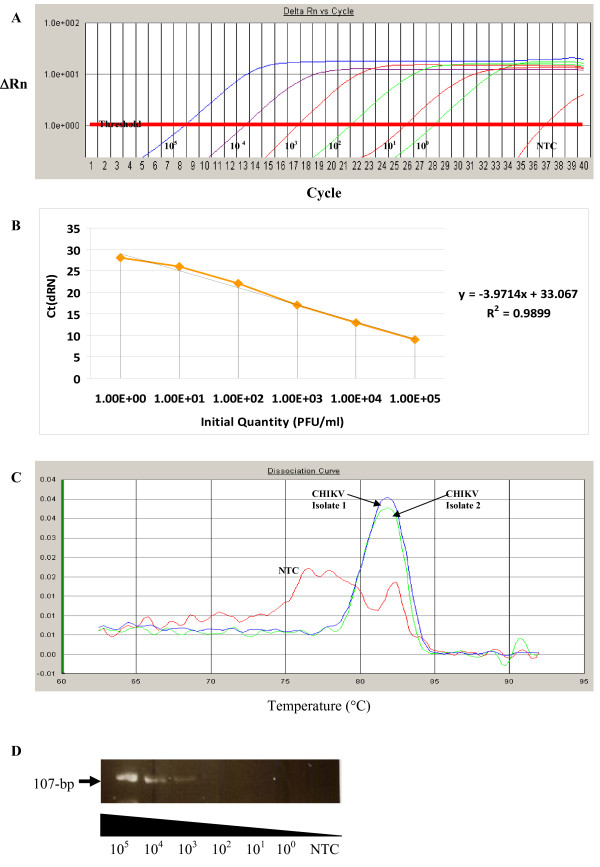
**One-step SYBR green-based RT-PCR for detection of CHIKV infection**. (A) Amplification profile and (B) the standard curve generated from the amplification profile of the one-step SYBR green-based quantitative RT-PCR of serially diluted CHIKV with known infective concentrations (10^0 ^to 10^5 ^PFU/ml) using the nsP2 primer set. A linear range of 6 logs of CHIKV dilution with a R^2 ^of 0.9899 is shown in B. (C) Melting curve analysis of the amplified product from two isolated strain of CHIKV using nsP2 primer set with a distinct melting peak (*T*_*m *_value) at 83°C. Primer-dimer can also be observed from the non-template control (NTC) with *T*_*m *_value below 76°C. (D) Semi-quantitative RT-PCR detection of serially diluted CHIKV with known infective concentrations (10^0 ^to 10^5 ^PFU/ml) using the nsP2 primer set.

Firstly, in order to construct a standard curve as well as to ascertain the possible detection limits of the one-step SYBR Green-based RT-PCR method using the specific nsP2 primer pairs in CHIKV detection, we tested 10-fold serial dilutions (10^5 ^- 10^0 ^PFU/ml) of seed viruses that had previously been quantitated by plaque forming assay. The amplification profile of the assay is shown in Figure 1A. The assay also showed linear results for the 6 logs of the serial dilutions of the seed viruses as indicated in Figure 1B. The detection limit of the specific nsP2 primer pair was calculated to be 1 PFU/ml for CHIKV. Since the binding of SYBR Green to DNA is sequence-independent and non-specific PCR fragments can also contribute to the fluorescent signal recorded by the instrument. A melting curve analysis was performed to confirm the presence of the specific nsP2 amplified DNA product which correlates with a distinct melting peak (*T*_*m *_value) at 83°C (Figure 1C) from two local isolated strains of CHIKV. Primer-dimer formation can often be detected in negative or weakly positive samples and the *T*_*m *_values of primer-dimers were found to be below 76°C (Figure 1C). To avoid non-specific signal detection of primer dimers, fluorescence for this assay will be acquired at 83°C and it was also noted that no primer-dimers were detected as demonstrated by melting curve analysis if optimal primer concentrations were followed.

In addition, the sensitivity and specificity of the SYBR Green based Real-Time RT-PCR was compared with another molecular gene amplification assay. A one-step semi-quantitative reverse transcriptase polymerase chain reaction (RT-PCR) was also performed with the same specific nsP2 primers (Table S1, Additional file 1.) using a commercial kit from Roche Diagnostics, Germany. As shown in Figure 1D, the semi-quantitative RT-PCR was highly specific in detecting the CHIKV RNA as indicated by the amplified product of 107-bp fragment but it was far less sensitive in the detecting the CHIKV RNA when compared to that of the one-step SYBR Green-based RT-PCR assay. The obvious amplified 107-bp DNA bands can only be observed to the detection limit of 10^3 ^PFU/ml of CHIKV.

**Figure 2:**
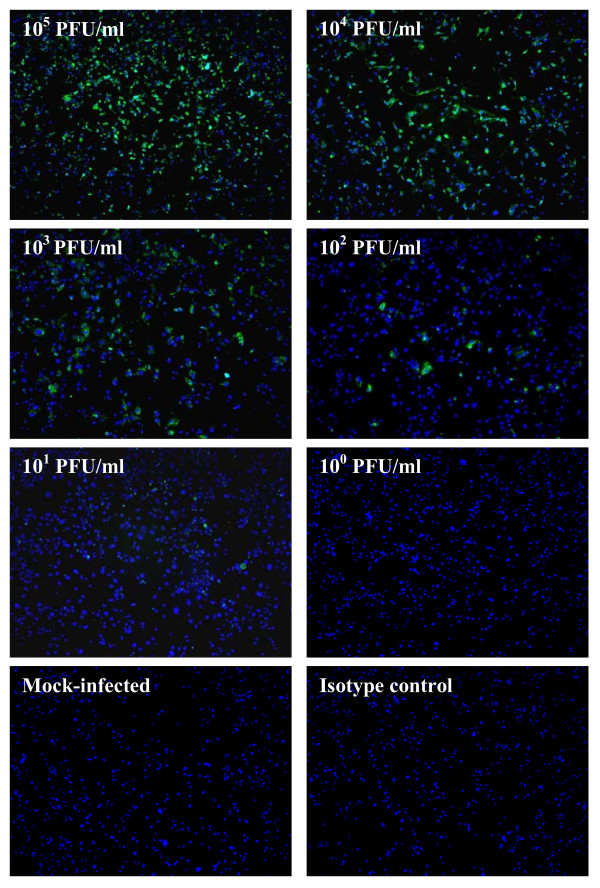
**Immunofluorescence assay (IFA) detection of viral antigen on CHIKV infected cells**. Vero cells are infected with serially diluted CHIKV (expressed as PFU/ml) for 2 days and subjected to immunofluorescence staining with anti-alphavirus antibody. The CHIKV-infected cells are stained green and the cell nuclei are stained blue with the nuclear stain, DAPI. Mock-infected control and isotype control (cells are stained with secondary anti-mouse antibody conjugated with FITC) are included to ensure the specificity of the assay.

Immunofluorescence assay (IFA) detection of viral antigen on CHIKV infected cells are routinely used by research as well as diagnostic laboratories due to the affordability and ease of experimental techniques [13-15]. We have also made a comparison of IFA with the one-step SYBR Green-based RT-PCR in the sensitivity of detection for CHIKV infection. Vero cells were first infected with 10-fold serial dilutions (10^5^-10^0 ^PFU/ml) of seed viruses that had previously been quantitated by plaque forming assay and subjected to immunofluorescence staining with primary antibody (anti-alphavirus, Santa Cruz Biotechnology) specific for CHIKV. As shown in Figure 2, the detection limit of IFA is limited to 10^2 ^PFU/ml of infectious virus titer. Despite the fact that IFA can be more affordable for routine detection of CHIKV infection as compared to RT-PCR, nevertheless, IFA can be limited by the sensitivity of the assay in the detection of CHIKV infection.

To verify the specificity of the nsP2 primers in the current one-step SYBR Green-based real-time RT-PCR, amplification of RNA extracted from uninfected human samples (sera samples, n = 5) and different RNA viruses were tested. No positive results were obtained from the uninfected human samples. Furthermore, no cross-reactivity was also observed for the closely related Ross River virus that belongs to the same the family Togaviridae as well as arboviruses [dengue viruses (serotype 1, serotype2, serotype 3 and serotype 4), kunjin virus, West Nile virus (Sarafend strain)], Enterovirus 71 and Echo virus type 7.

**Figure 3:**
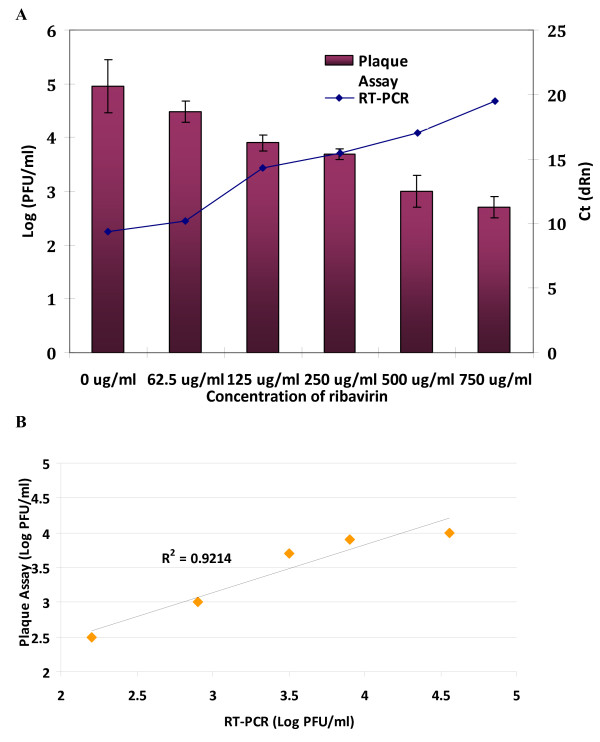
**Inhibitory assay of CHIKV infection using ribavirin**. (A) Dosage dependent inhibition of CHIKV in Vero cells treated with different concentrations of ribavirin (62.5 μg/ml to 750 μg/ml). The cellular supernatants containing the infectious virus titers are determined by plaque assays (expressed as Log PFU/ml) as well as the one-step SYBR green-based quantitative RT-PCR (expressed as Ct value). (B) Correlation between the viral titers expressed as Log PFU/ml obtained from plaque assays and the one-step SYBR green-based quantitative RT-PCR are shown. Results from three independent experiments are included and statistical analysis is performed with a non-parametric (Spearman's correlation) test.

To validate the applicability of this CHIKV one-step Green-based real-time RT-PCR for quantitative measurements, we went on further to perform parallel determinations by the one-step SYBR Green-based RT-PCR and viral infectivity titration via virus plaque assay in these experiments of inhibition of virus replication by ribavirin in vitro. Ribavirin is a member of the nucleoside anti-metabolite drugs that interfere with duplication of viral genetic material [16] and has been reported to have anti-viral activity against several members of the genus Alphavirus including CHIKV [17]. Vero cells were first infected with CHIKV at a M.O.I. of 1 for 1 hour and treated for 48 hours with non-cytotoxic concentrations of ribavirin (62.5 μg/ml, 125 μg/ml, 250 μg/ml, 500 μg/ml and 750 μg/ml). The cytotoxicity assay was carried out using MTT assay (Chemicon). Virus yield from the supernatants of treated cells was determined by both assays (one-step RT-PCR or plaque assays) after 48 hours of infection. As shown in Figure 3A, dosage-dependent inhibition of virus yield was observed with increasing concentration of ribavirin treatment as indicated by both viral infectivity (plaque assays) and viral RNA (one-step RT-PCR with increasing Ct values at higher drug concentrations). As revealed in Figure 3B, we can also show good direct correlation (R^2 ^= 0.9214) between viral titers of the two different quantification methods obtained from three independent experiments. The Log PFU/ml data for the one-step RT-PCR are calculated from the standard curve of Ct values versus the virus quantity (PFU/ml) as indicated in Table 1 of the titrated seed CHIKV.

**Table 1:** Sensitivity and Specificity of the SYBR Green-based real-time RT-PCR using CHIKV nsP2 primer set.

Sample	Quantity (PFU/ml)	Ct Value	*Assay Results*
Titrated seed CHIKV			
Undiluted	10^5^	9.11	*Positive*
Diluted 10^-1^	10^4^	13.75	*Positive*
Diluted 10^-2^	10^3^	17.89	*Positive*
Diluted 10^-3^	10^2^	22.00	*Positive*
Diluted 10^-4^	10^1^	26.45	*Positive*
Diluted 10^-5^	10^0^	28.64	*Positive*
Other viruses tested in this assay			
Ross River virus (Alphavirus)	10^4^	40.00	*Negative*
Dengue virus Serotype 1 (Flavivirus)	10^4^	40.00	*Negative*
Dengue virus Serotype 2 (Flavivirus)	10^5^	40.00	*Negative*
Dengue virus Serotype 3 (Flavivirus)	10^4^	40.00	*Negative*
Dengue virus Serotype 4 (Flavivirus)	10^4^	40.00	*Negative*
Kunjin virus (Flavivirus)	10^5^	40.00	*Negative*
West Nile virus (Sarafend, Flavivirus)	10^6^	40.00	*Negative*
Enterovirus 71 (Piconavirus)	10^6^	40.00	*Negative*
Echo virus type 7 (Piconavirus)	10^6^	40.00	Negative

The application of PCR in molecular diagnosis has gradually replaced traditional cell culture method as the gold standard for virus detection. The real-time PCR assays have many advantages comparing to conventional PCR including simplicity, rapidity, sensitivity, and low contamination. In this study, the SYBR green I-based RT-PCR for the quantitation and detection of CHIKV is described. The assay is linear over six orders of magnitude and requires approximately 2 hours from sample preparation to data analysis. The one-step assay further adds convenience and minimizes sample handling, which may cause cross-contaminations and decreases quantitative reliability. The SYBR Green-based RT-PCR assay is known to be less specific than the TaqMan RT-PCR. However, it has the advantages of simplicity in primer design and universal RT-PCR protocols suitable for multiple target sequences. In this study, we have also found that the optimization of primer concentration is critical in preventing primer-dimers and non-specific amplification of other unrelated gene products. The sensitivity of the one-step SYBR green I-based RT-PCR for detection of CHIKV infection was highlighted when comparison was made to conventional semi-quantitative RT-PCR assay as well as traditional IFA detection assay. Furthermore, the current RT-PCR assay is highly specificity and is able to differentiate closely related Ross River virus from Chikungunya virus, which both viruses belongs to the same genus of *Alphavirus *in the family of *Togaviridae*. This quantitative method of one-step SYBR green I-based RT-PCR for detection of CHIKV infection was further validated by *in vitro *experiments in which ribavirin, a well-known RNA virus inhibitor, showed a dose-dependent inhibition of virus replication, assessed by viral infectivity and viral RNA production. Together, these data strongly suggest that this current method can be a useful tool for rapid detection and quantification of CHIKV during natural infection, in research laboratory settings and possibly monitoring the extent of viral replication in patients for clinical diagnosis and epidemiological surveillance of possible emerging epidemic of CHIKV infection.

## Competing interests

The authors declare that they have no competing interests.

## Authors' contributions

JJHC and PSH designed research; PSH and JJHC performed research; PSH, MLN and JJHC analyzed data and wrote the paper. All authors have read and approved the final manuscript.

## Supplementary Material

Additional file 1 CHIKV nsP2 gene primer set and nsP2 gene sequence homology of different CHIKV strains. Click here for file
