# Supplementary material for: Establishment of one-step SYBR green-based real time-PCR assay for rapid detection and quantification of chikungunya virus infection
Source: Virol J. 2010 Jan 21;7:13. doi: 10.1186/1743-422X-7-13 (PMC2827404; doi:10.1186/1743-422X-7-13)
Supplement: Additional file 1 — CHIKV nsP2 gene primer set and nsP2 gene sequence homology of different CHIKV strains. [file 1743-422X-7-13-S1.DOC]

**Table S1. CHIKV nsP2 gene primer set and nsP2 gene sequence homology of different CHIKV strains.**

nsP2 Forward Primer

nsP2 Reverse Primer

Malaysia


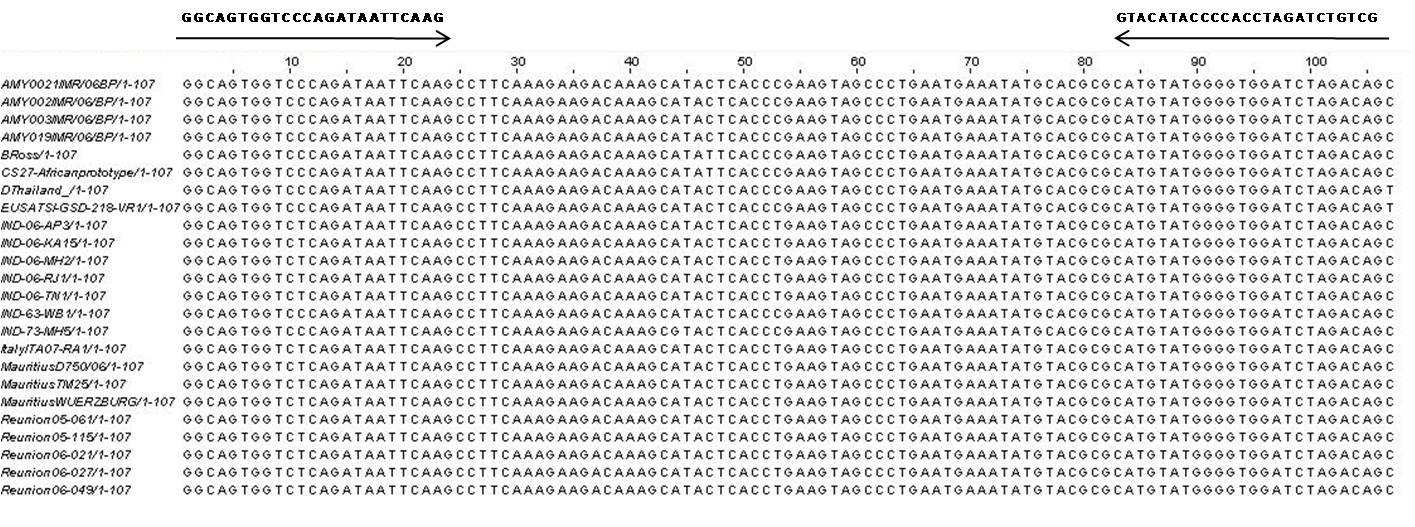


India
